# Acknowledgement to Reviewers of *Journal of Intelligence* in 2018

**DOI:** 10.3390/jintelligence7010001

**Published:** 2019-01-08

**Authors:** J. Intell. Editorial Office

**Affiliations:** MDPI, St. Alban-Anlage 66, 4052 Basel, Switzerland; jintelligence@mdpi.com

Rigorous peer-review is the corner-stone of high-quality academic publishing. The editorial team greatly appreciates the reviewers who contributed their knowledge and expertise to the journal’s editorial process over the past 12 months. In 2018, a total of 48 papers were published in the journal, with a median time to first decision of 29 days and a median time to publication of 69 days. The editors would like to express their sincere gratitude to the following reviewers for their cooperation and dedication in 2018:

Abele, StephanHerzberg, Philipp Y.Nußbeck, FridtjofAhmetoglu, GorkanHilbert, SvenOshio, AtsushiAmbrose, DonHolt, DanielPanno, AngeloAnderson, MikeHülür, GizemPlucker, JonathanAschenbrenner, AndrewIorga, MagdalenaRandall, JasonAtit, KinnariJain, PinkyRanger, JochenAusloos, MarcelJayawickreme, ErandaRauthmann, JohnBadger, JuliaJonason, Peter K.Redifer, JenniBaron, JonathanKalalahti, MiraRey-Mermet, AlodieBeauducel, AndreKanaya, TomoeRinaldi, LucaBeaujean, AlexanderKarwowski, MaciejRindermann, HeinerBergold, SebastianKaufman, JamesRuiz-Barquín, RobertoBoom, JanKhan, Atif AliSajjadi, PejmanBratko, DenisKievit, RogierSchmidt, FrankBroniatowski, David AndreKlimes-Dougan, BonnieSchmiedek, FlorianBrouwer, RachelKlose, DianaSchmitz, FlorianCabeza De Baca, TommyKo, CelineSchneider, W. JoelCaemmerer, Jacqueline M.Kornhaber, MindySchroeders, UlrichCampbell, Frances A.Kovacs, KristofSchubert, Anna-LenaChuderski, AdamKretzschmar, AndreSchweizer, KarlColom, RobertoKretzschmar, AndréShelton, NicoleConway, AndrewKyllonen, PatrickSimonton, Dean KeithCorbin, Jonathan CharlesLakin, Joni M.St Clair-Thompson, HelenCosta, PaulLaming, Donald Richard JohnStanovich, KeithCostello, ShaneLapalme, MatthewStaudinger, Ursula M.Coyle, Thomas R.Lautrey, JacquesSternberg, Robert J.Dauvier, BrunoLechner, ClemensStorek, JosephineDe Boeck, PaulLemaire, PatrickStough, LonDemetriou, AndreasLeon Rubio, Jose MariaSvob Strac, DubravkaDetterman, DouglasLesiuk, TeresaTe Nijenhuis, JanDiaz-Sarachaga, Jose ManuelLoi, NatashaToplak, MaggieDijk, Marijn VanLuis Ubago, JoseUttal, DavidDresp-Langley, BirgittaMarx, PeterVan Der Linden, DimitriDunkel, CurtisMarzocchi, Gian MarcoVan Der Maas, Han L.J.Fasko, DanielMarzoli, DanieleVon Stumm, SophieFayn, KirillMatta, MichaelWacker, JanFernandez, SébastienMattison, RichardWai, JonathanFigueredo, Aurelio JoséMayer, AxelWakeman, ShawneeFontaine, JohnnyMcAbee, SamuelWaterhouse, LynnForgeard, MarieMeara, PaulWatkins, Marley W.Frey, MeredithMichalkiewicz, MarthaWebb, Rose MaryGardner, HowardMottus, ReneWeldon, RebeccaGoethals, GeorgeMurphy, KevinWenos, JeanneGoldhammer, FrankMurphy, RaeganWeststrate, NicGolle, JessikaNaglieri, Jack A.Wood, DustinGonzález Valero, GabrielNarvaez, DarciaWoodley, MichaelGraesser, ArthurNauta, NoksZelazo, Philip DavidGrossmann, IgorNęcka, EdwardZiegler, MatthiasHancock, Peter J.B.Neubauer, AndreasZou, LiyeHein, Sascha DNúñez Pérez, José CarlosZu, Jiyun

